# Social and housing indicators of dengue and chikungunya in Indian adults aged 45 and above: Analysis of a nationally representative survey (2017-18)

**DOI:** 10.1186/s13690-022-00868-5

**Published:** 2022-04-20

**Authors:** Winnie Paulson, Naveen Kumar Kodali, Karuppusamy Balasubramani, Rashi Dixit, Savitha Chellappan, Sujit Kumar Behera, Praveen Balabaskaran Nina

**Affiliations:** 1 Department of Epidemiology and Public Health, School of Life Sciences, Central University of Tamil Nadu, Tiruvarur, India; 2 Department of Geography, School of Earth Sciences, Central University of Tamil Nadu, Tiruvarur, India; 3 Indian Council of Medical Research- National Institute of Traditional Medicine, Belagavi, India

**Keywords:** LASI, Social determinants of dengue, Social determinants of chikungunya, Risk factors of dengue and chikungunya

## Abstract

**Background:**

Dengue and chikungunya (CHIKV) are the two major vector-borne diseases of serious public health concern in India. Studies on socioeconomic and housing determinants of dengue and CHIKV at a pan-India level are lacking. Here, we took advantage of the recently carried out Longitudinal Ageing Study in India (LASI) carried out across all the states and Union Territories of India to study the social indicators of dengue and CHIKV in India.

**Methods:**

LASI-1 (2017-2018) data on the self-reported period prevalence of dengue and CHIKV from 70,932 respondents aged ≥45 years were used for this analysis. The state-wise distribution of dengue and CHIKV was mapped. Prevalence was estimated for each study variable, and the difference was compared using the χ2 test. The adjusted odds ratios (AOR) of the socioeconomic and housing variables for dengue and CHIKV were estimated using the multiple logistic regression model.

**Results:**

Urban residence is the major socio-economic indicator of dengue and CHIKV (dengue AOR: 1.57, 95% CI: 1.18-2.11; CHIKV AOR: 1.84, 95% CI: 1.36-2.49). The other notable indicator is wealth; rich respondents have higher odds of dengue and CHIKV. Adults older than 54 years and those with high school education and above are associated with a lower likelihood of dengue and CHIKV. In addition, CHIKV is associated with scheduled and forward castes, households with improper toilet facilities, open defecation, and kutcha house type.

**Conclusions:**

Despite the limitation that the data is only from adults ≥ 45, this analysis provides important insights into the socioeconomic and housing variables associated with higher odds of dengue and CHIKV in India. Understanding these determinants may assist in the national planning of prevention and control strategies for dengue and CHIKV.

**Supplementary Information:**

The online version contains supplementary material available at 10.1186/s13690-022-00868-5.

## Background

The dengue virus, primarily transmitted by mosquito species *Aedes aegypti*, and to a lesser extent by *Ae. albopictus* consists of four serotypes (DENV1-4), which contribute to the distinct epidemiological spread of dengue [1]. In recent decades, the global incidence of dengue has increased alarmingly, and about half the world’s population are at risk [1]. Globally, there is an eight-fold increase in dengue cases from 505,430 in 2000 to 5.2 million cases in 2019 [1]. Even though dengue risk is reported in 128 countries, the actual burden (70%) is in Asia, and India is one of the major contributors [2, 3].

In India, from 1990 onwards, there have been frequent dengue epidemics [4]. Compared to 1998-2009 (82,327 cases), dengue cases have increased by a factor of ~2.6 in 2010-14 (213,607 cases) [5]. In India, the actual numbers of dengue could be grossly underreported [6, 7], as the majority of the cases are mild/asymptomatic and/or misdiagnosed [8]. In 2017, a nationwide dengue serosurvey carried out across 60 districts in 15 states of India, covering five geographical regions reveal a seroprevalence of 48·7% (95% CI: 43·5-54·0); the highest positivity rate (56.2%, 95% CI: 49.0-63.1) is seen in 18-45 years old [7]. This study, based on the constant force of infection models, also estimates ~13 million dengue infections across the 30 Indian states [7]. Initially restricted to urban areas, dengue has spread to rural regions of India, making the entire country susceptible [4, 9].

In India, Chikungunya (CHIKV) is the second major vector-borne viral disease transmitted by *Ae. aegypti* and *Ae. albopictus*. India has witnessed CHIKV outbreaks from 1963-74 [10]. This was followed by three decades of quiescence, and CHIKV re-emerged in 2005 with 1.39 million suspected cases in 2006, and after a gradual decline till 2014, the cases started rising in 2015, with 67,769 cases reported in 2017 [11]. A nation-wide CHIKV serosurvey (2017-18) of 15 states showed a seroprevalence of 18.1% (95% CI: 14·2-22·6), and southern region was highest (43.1%; 95% CI: 34.3-52.3) [12]. Furthermore, seroprevalence was much higher in urban areas (40.2%; 95% CI: 31.7-49.3) compared to rural (11.5%; 95% CI: 8.8-15) [12]. An estimated 56.3-98% of the population in India are still susceptible to CHIKV, and this could explain the continuous transmission of CHIKV after re-emerging in 2005 [12].

Indian Council of Medical Research and Department of Health Research have set up Viral Research Diagnostic Laboratories throughout India to diagnose viral diseases [13]. The major vector control intervention for dengue and CHIKV in India are source reduction, larviciding the positive containers (Temephos 50% EC), indoor space spray (Pyrethrum, Cyphenothrin 5% EC) and outdoor fogging (Technical Malathion, Cyphenothrin 5% EC) [11].

In India, dengue and CHIKV pose a serious public health risk, and for effective control strategies, in addition to environmental risk factors, it is important to understand the socioeconomic determinants of health (SDH) influencing transmission. Income, education, employment status, housing and access to affordable health care services are some of the important SDH that affect health equity [14]. Studies detailing the socioeconomic and housing risk factors of viral vector-borne diseases are sparse in India and are largely focused on selected districts [15–18]. A pan-India study on the socioeconomic and housing indicators of vector-borne diseases may provide important insights into their prevention and control. A nationwide Longitudinal Ageing Study in India (LASI) wave 1 was carried out for the first time in India (2017-18) to collect important information on health, health care, socioeconomic status (SES) and self-reported prevalence of vector-borne diseases, including dengue and CHIKV among adults aged 45 and above [19]. Here, we have analysed the LASI data and detailed the SES and housing risk factors of dengue and CHIKV.

## Methods

### Data and participants

Data from the recent LASI wave 1 (2017-2018) carried out by the International Institute for Population Sciences (IIPS), Mumbai, India, was used for the analysis. The LASI wave 1 is a nationally representative study of all states and Union Territories (UT) except Sikkim. The survey gathered vital information on health, infectious diseases, socioeconomic determinants, and consequences of population ageing from 72,252 individuals. A multistage clustering sampling design was adopted to obtain the data from the non-institutional residents aged ≥45 years and their spouses (regardless of age). In the first stage of the sampling process, primary sampling units, i.e. sub-districts (Tehsils/Talukas), were selected in each state/UT. During the second stage, villages (rural areas) and wards (urban areas) were selected from all the primary sampling units and in the third stage, households and individuals were selected. The sampling procedure was extended by one more step in the urban areas where a census enumeration block was chosen before selecting households. LASI individual and household datasets were merged to maximise the study’s objectives. The merging of the datasets resulted in missing values, which were less than 2%. The final sample size analysed was 70,932, out of which 30,283 respondents (40.7%) were between 45-54 years, 28,456 (40.4%) were 55-69 years, and 12,193 (18.9%) were ≥70 years. Of the total respondents, 57.8% were females, and 69% were from rural areas. Participants have provided written consent to participate in the survey. LASI data were obtained after a written request to IIPS [19].

### Study variables

#### Outcome variable

The presence of dengue and CHIKV in a household was identified based on the following questions: 1. In the past two years, have you had dengue? 2. Were you treated by health professionals for dengue? 3. In the past two years, have you had CHIKV? 4. Were you treated by health professionals for CHIKV? The options were ‘Yes’ and ‘No’. Those respondents who had both dengue and CHIKV and were treated by a health professional were considered a case of dengue and CHIKV. The responses were coded as a binary variable (‘0’for absence and ‘1’ for presence), and the total respondents with dengue and CHIKV cases are 607 and 1358, respectively.

#### Household variables

The household variables utilized are household size (1-5 or ≥6 members), type of house: permanent (pucca/semi-pucca) or temporary (kutcha), location of water source (own dwelling, yard/plot or outside dwelling), toilet type (flushed to piped sewer system/septic tank/pit latrine, pit latrine/twin pit/composting toilet and open defecation), cooking fuel (clean fuel: LPG, biogas and electricity; unclean fuel: kerosene, charcoal, coal, crop residue, wood/shrub and dung cake) and having a damp wall or ceiling (yes/no).

#### Socioeconomic status variables (SES)

The SES variables used for this analysis are age-group (45-54 years, 55-69 years and ≥70 years), sex (male/female), residence (rural/urban), income category (poorest, poorer, middle, richer and richest - based on Monthly Per Capita Consumption Expenditure (MPCE) quintiles), education (0 school years, 1-5 school years, 6-12 school years and college and higher), Caste (Scheduled Castes [SC], Scheduled Tribes [ST], Other Backward Classes [OBC] and forward castes category), and occupation (not working, agricultural and allied, self-employed and wage/salary worker). The household and socioeconomic variables were selected based on previous studies [20–23].

#### Statistical analysis

Frequency and percentage distribution tables were prepared for all the variables used in this study. Dengue and CHIKV prevalence with each housing condition and SES variables were estimated, and the difference was compared using the χ2 test. The variables for multiple logistic regression analyses were chosen based on the purposeful selection of variables [24]. Following univariable analyses, predictors with <0.25 significance level were selected for the multivariable model. In this model, variables not significant at the 0.10 level were removed one at a time, and its effect on the odds ratios of other variables (15% change) were assessed. In the last step, those variables that did not meet the criterion of p-value less than 0.25 during the univariable analyses were added to the model. The predictors were retained if the p-value was less than 0.10 or their addition caused a change of 15% or more in the odds ratios for at least one of the categories of the variables*.* Collinearity was checked for the predictors, and variance inflation factor values were <5. We have applied sampling weights provided in the LASI datasets during the analysis to obtain reliable statistical estimates. STATA 16 (StataCorp LLC, College Station, Texas, USA) statistical software was used for the data analysis. We followed the Strengthening the Reporting of Observational Studies in Epidemiology (STROBE) guidelines for reporting the survey data (https://www.strobe-statement.org/checklists/). The STATA Do file in txt format is given as **Additional file 1.**

#### Spatial analysis

The state-wise prevalence (%) was used to visualize the spatial distribution of dengue and CHIKV using the ArcGIS 10.4 software (https://desktop.arcgis.com). The dengue/CHIKV prevalence of individual state/UT was analysed and grouped into four classes, two above and two below the national prevalence. The choropleth technique was used for visualization, where the darker hue was used to denote higher prevalence.

## Results

### Prevalence of dengue and CHIKV in adults ≥45 years

**Fig. 1:**
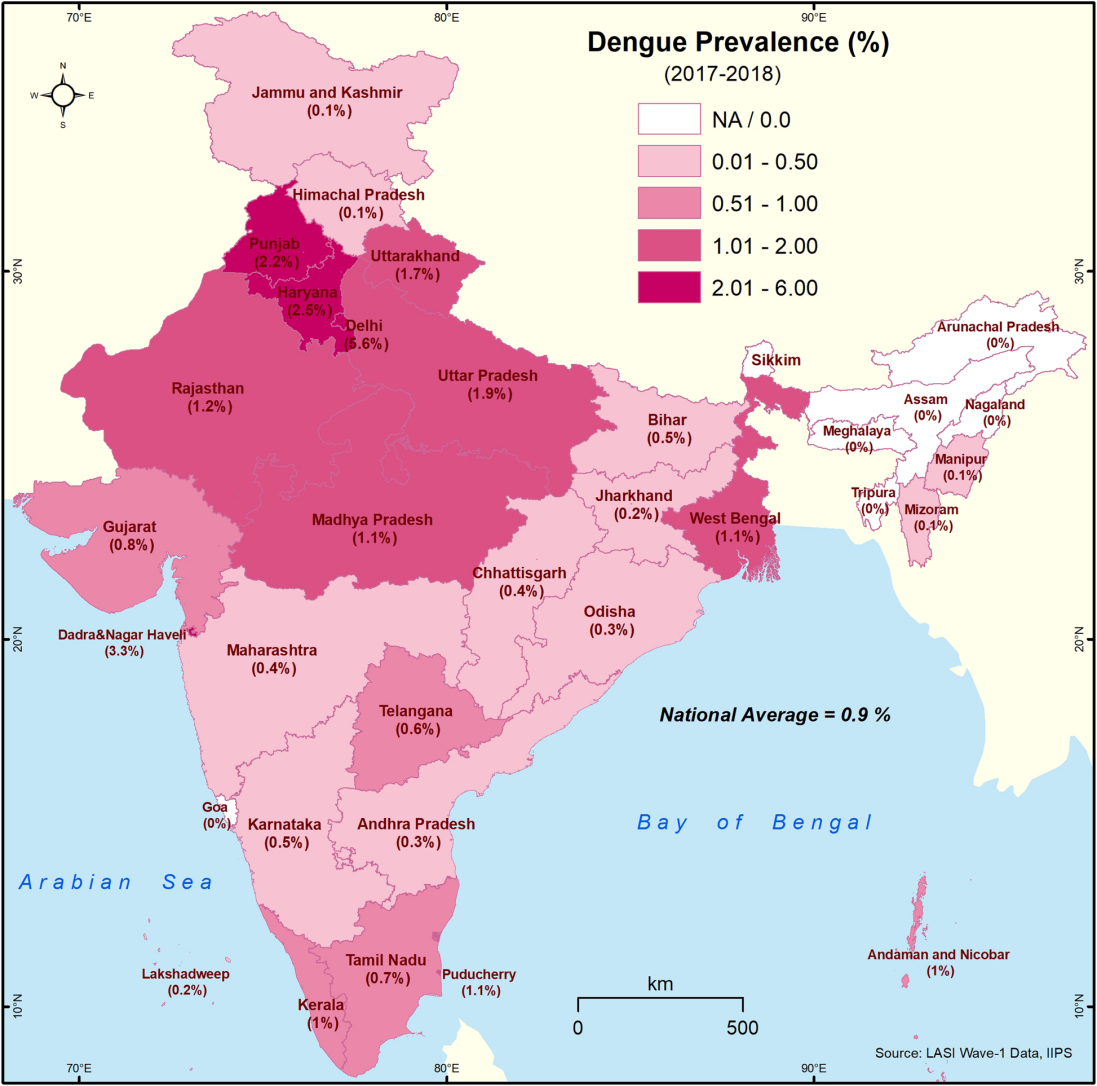
Self-reported prevalence of dengue in different states and Union Territories of India (LASI-1 2017-2018). The intervals represent dengue prevalence. The darker the shade, higher is the prevalence

**Fig. 2:**
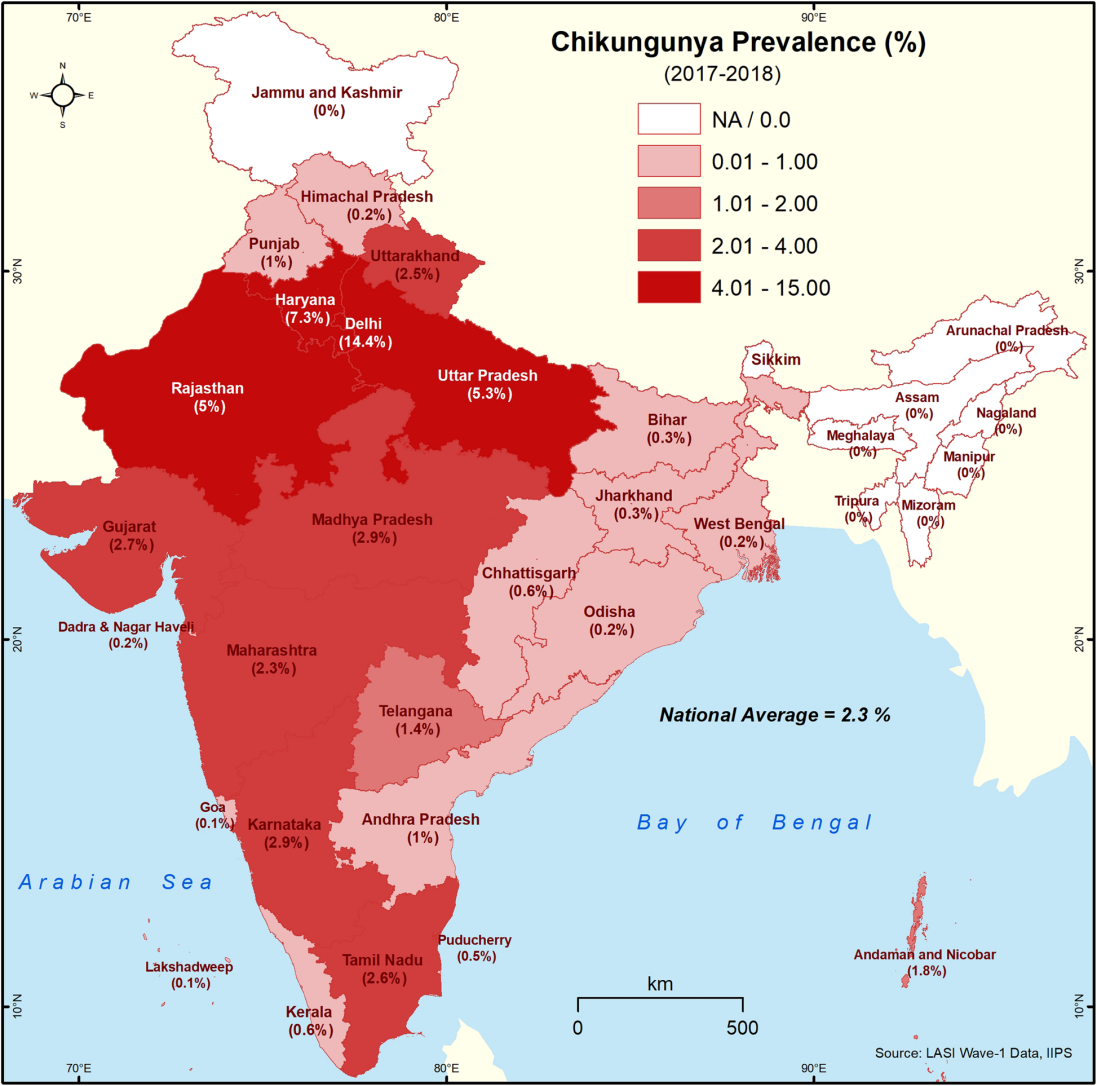
Self-reported prevalence of CHIKV in different states and Union Territories of India (LASI-1 2017-2018). The intervals represent CHIKV prevalence. The darker the shade, higher is the prevalence

The distribution of all the study variables is shown in Table 1. The period prevalence of dengue and CHIKV is 0.87% (95% CI: 0.77-0.99%), and 2.29% (95% CI: 2.11-2.49%) respectively. Figs. 1-2 show the distribution of dengue and CHIKV across India’s states/UT. Dengue is highly prevalent in the northern states of India, and the highest prevalence is observed in Delhi (5.6%), followed by Dadra & Nagar Haveli (3.3%) and Chandigarh (3.1%). Delhi (14.3%) and Haryana (7.3%) show the highest prevalence of CHIKV cases. Dengue/CHIKV was not reported in most north-eastern states.

**Table 1:** Distribution of socioeconomic and housing variables in older adults in India, LASI-1 (2017-2018)

Variables	%	Total
Chikungunya	2.29	1358
Dengue	0.87	607
**Age Group**		
45-54 years	40.68	30,283
55-69 years	40.45	28,456
≥ 70 years	18.87	12,193
**Sex**		
Female	57.83	40,867
Male	42.17	30,065
**Residence**		
Rural	69.25	45,911
Urban	30.75	25,021
**MPCE Quintile**		
Poorest	20.94	13,879
Poorer	21.26	14,243
Middle	20.17	14,261
Richer	19.51	14,421
Richest	18.11	14,128
**Education**		
0 School years	49.64	32,652
1-5 School years	17.45	12,815
6-12 School years	26.73	21,316
college & above	6.17	4,148
**Caste**		
Scheduled tribe	19.98	11,885
Scheduled caste	8.90	12,264
Other backward castes	46.25	26,697
Forward caste	24.87	17,505
**Occupation**		
Not Woking	50.11	36,388
Agricultural and Allied	28.12	18,260
Self-employed /Wage/Salary	21.77	16,179
**Household Size**		
1-5 members	63.04	45,234
≥ 6 members	36.96	25,698
**House Type**		
Pucca/Semi Pucca	83.25	57,735
Kutcha	16.75	12,813
**Water Source**		
Own dwelling	47.06	33,489
Own yard/plot	21.66	15,709
Outside dwelling	31.28	17,903
**Toilet type**		
Flush type	47.67	36,951
Pit latrine/twin pit/composting	25.65	20,139
Open defecation	26.67	13,587
**Cooking fuel**		
Clean	52.30	38,243
Unclean	47.70	32,434
**Damp wall/ceiling**		
Yes	79.03	56,209
No	20.97	14,468

Total is unweighted, and percentages are weighted

The prevalence of dengue and CHIKV associated with SES and housing factors in adults ≥45 years is shown in Table 2. Dengue and CHIKV are less in males (0.74% and 2.05%, respectively) compared to females (0.97% and 2.47%, respectively). Dengue and CHIKV are higher in urban (1.12% and 2.91%, respectively) than in rural areas (0.76% and 2.02%, respectively). A slightly higher prevalence of dengue and CHIKV was seen in the rich. ST had the lowest prevalence of dengue (0.55%) and CHIKV (1.17%). The prevalence of dengue was highest in SC (1.12%), and CHIKV was highest in the forward caste (2.94%). The prevalence of both diseases is higher in pucca/semi-pucca houses. The prevalence of CHIKV is lowest in households that have flush toilets. For dengue and CHIKV, the prevalence was higher in households using clean fuel. Households with a damp wall/ceiling have a lower prevalence of CHIKV when compared to ones without a damp wall/ceiling (2.07% *vs* 3.09%).

**Table 2:** Prevalence of dengue and chikungunya by socioeconomic and housing variables in older adults in India, LASI-1 (2017-2018)

	Dengue %			χ^2^	Chikungunya %			χ^2^
Age Group	No	Yes	95% CI	p value	No	Yes	95% CI	p value
45-54 years	98.99	1.01	[0.86-1.18]		97.46	2.54	[2.21-2.91]	
55-69 years	99.23	0.77	[0.62-0.95]	0.207	97.74	2.26	[2.01-2.54]	
≥70 years	99.19	0.81	[0.54-1.21]		98.15	1.85	[1.53-2.23]	0.028
**Sex**								
Female	99.03	0.97	[0.83-1.14]		97.53	2.47	[2.21-2.77]	
Male	99.26	0.74	[0.60-0.91]	0.043	97.95	2.05	[1.82-2.30]	0.021
**Residence**								
Rural	99.24	0.76	[0.64-0.91]		97.98	2.02	[1.84-2.22]	
Urban	98.88	1.12	[0.94-1.33]	0.002	97.09	2.91	[2.50-3.39]	0.000
**MPCE Quintile**								
Poorest	99.32	0.68	[0.52-0.89]		98.23	1.77	[1.47-2.12]	
Poorer	99.19	0.81	[0.62-1.06]		97.93	2.07	[1.76-2.44]	
Middle	99.22	0.78	[0.59-1.04]		97.48	2.52	[1.98-3.19]	
Richer	98.98	1.02	[0.78-1.33]	0.091	97.48	2.52	[2.15-2.96]	
Richest	98.88	1.12	[0.82-1.51]		97.33	2.67	[2.28-3.11]	0.019
**Education**								
No school years	99.04	0.96	[0.82-1.13]		97.54	2.46	[2.16-2.79]	
1-5 school years	99.13	0.87	[0.60-1.26]		97.91	2.09	[1.78-2.46]	
6-12 school years	99.20	0.80	[0.63-1.02]		97.84	2.16	[1.88-2.48]	
college & above	99.53	0.47	[0.27-0.84]	0.192	97.88	2.12	[1.54-2.91]	0.319
**Caste**								
Scheduled caste	98.88	1.12	[0.85-1.48]		97.36	2.64	[2.08-3.35]	
Scheduled tribe	99.45	0.55	[0.33-0.92]		98.83	1.17	[0.86-1.60]	
Other backward	99.22	0.78	[0.64-0.94]	0.036	97.90	2.1	[1.89-2.34]	
Forward caste	99.04	0.96	[0.76-1.21]		97.06	2.94	[2.56-3.37]	0.000
**Occupation**								
Non-working	98.97	1.03	[0.85-1.23]		97.76	2.24	[2.02-2.49]	
Agriculture and allied	99.47	0.53	[0.41-0.69]		97.63	2.37	[2.06-2.71]	
Self-employed or wages	99.03	0.97	[0.79-1.19]	0.000	97.69	2.31	[1.82-2.93]	0.862
**Household Size**								
0-5	99.12	0.88	[0.75-1.04]		97.74	2.26	[2.02-2.53]	
≥ 6	99.14	0.86	[0.71-1.04]	0.804	97.65	2.35	[2.08-2.65]	0.655
**House type**								
Pucca/semi Pucca	99.08	0.92	[0.81-1.05]		97.51	2.49	[2.28-2.72]	
kutcha	99.39	0.61	[0.37-1.01]	0.113	98.71	1.29	[1.00-1.67]	0.000
**Water source**								
Own dwelling	98.97	1.03	[0.87-1.21]		97.38	2.62	[2.37-2.91]	
Own yard/plot	99.47	0.53	[0.38-0.75]		98.65	1.35	[1.10-1.66]	
outside dwelling	99.09	0.91	[0.70-1.18]	0.006	97.48	2.52	[2.10-3.01]	0.000
**Toilet type**								
Flush type	99.12	0.88	[0.73-1.07]		98.00	2.00	[1.79-2.23]	
Pit latrine /twin pit/composting	99.01	0.99	[0.78-1.25]		97.36	2.64	[2.32-3.00]	
Open defecation	99.25	0.75	[0.58-0.96]	0.289	97.54	2.46	[2.01-3.01]	0.023
**Cooking fuel**								
Clean	99.03	0.97	[0.83-1.13]		97.52	2.48	[2.26-2.71]	
Unclean	99.23	0.77	[0.62-0.96]	0.100	97.92	2.08	[1.78-2.41]	0.048
**Damp wall/ceiling**								
Yes	99.10	0.90	[0.78-1.04]		97.93	2.07	[1.90-2.26]	
No	99.23	0.77	[0.61-0.97]	0.258	96.91	3.09	[2.54-3.76]	0.000

Total is unweighted, and percentages are weighted

### Relationship between the SES and housing variables and dengue/CHIKV in adults ≥45 years

The odds ratios of dengue and CHIKV for the SES and housing variables are shown in Table 3. Urban residents and the rich have higher odds of dengue. Adults aged over 54 years, respondents with more than six school years, those in agriculture or allied fields and households with water-source in their own yard/plot have lower odds of dengue. Urban residents have 1.6 times (AOR: 1.57; 95% CI: 1.18-2.11) higher odds for dengue than rural residents. Those from the highest-income quintile have higher odds of dengue than those who are from the poorer quintile (AOR: 2.10; 95% CI: 1.34-3.31). Adults older than 54 years have a lower likelihood of dengue than adults between 45-54 years. Compared to those with no school education, the lowest risk (AOR: 0.24; 95% CI: 0.12-0.49) for dengue was seen in the college-educated. Those in agriculture or allied fields have lower odds of dengue than non-working individuals. Households with water-source in their yard/plot have lower odds (AOR: 0.55; 95% CI: 0.37-0.80) for dengue than households with water-source in their dwelling.

**Table 3:** Odds ratios of dengue and chikungunya by socioeconomic and housing variables in older adults in India, LASI-1 (2017-2018)

Variables	Dengue			Chikungunya		
Age Group	UOR	OR	95% CI	UOR	OR	95% CI
45-54 years	1	1		1	1	
55-69 years	0.76*	0.69**	0.53 – 0.91	0.89	0.87	0.72 – 1.04
≥ 70 years	0.80	0.65	0.42 – 1.02	0.72*	0.71**	0.55 – 0.90
**Sex**						
Female	1			1		
Male	0.76*			0.82*		
**Residence**						
Rural	1	1		1	**1**	
Urban	1.47*	1.57**	1.18 – 2.11	1.45**	1.84**	1.36 – 2.49
**MPCE Quintile**						
Poorest	1	1		1	1	
Poorer	1.19	1.36	0.93 – 2.01	1.17	1.34	1.03 – 1.75
Middle	1.15	1.34	0.89 – 2.01	1.43*	1.65**	1.17 – 2.32
Richer	1.50*	1.81**	1.23 – 2.68	1.44**	1.72**	1.31 – 2.26
Richest	1.65*	2.10**	1.34 – 3.31	1.52**	1.92*	1.44 – 2.56
**Education**						
0 School years	1	1		1	1	
1-5 School years	0.91	0.81	0.52 – 1.27	0.85	0.80*	0.64 – 0.99
6-12 School years	0.83	0.58**	0.41 – 0.83	0.88	0.70**	0.57 – 0.86
college & above	0.49*	0.24**	0.12 – 0.49	0.86	0.56**	0.38 – 0.85
**Caste**						
Scheduled tribe	1			1	1	
Scheduled caste	2.06*			2.28**	1.89**	1.28 – 2.81
Other backward caste	1.43			1.81*	1.51*	1.05 – 2.16
Forward caste	1.77			2.55**	2.24**	1.55 – 3.26
**Occupation**						
Not a current worker	1	1		1		
Agricultural and allied	0.52**	0.54**	0.39 – 0.77	1.06		
Self-employed /Wages	0.94	0.90	0.67 – 1.20	1.03		
**Household-Size**						
1-5 members	1			1		
≥ 6 members	0.97			1.04		
**House Type**						
Pucca/Semi Pucca	1			1	1	
Kutcha	0.66			0.51**	0.50**	0.37 – 0.67
**Water Source**						
Own dwelling	1	1		1	1	
Own yard/plot	0.51**	0.55**	0.37 – 0.80	0.51**	0.57**	0.45 – 0.72
Outside dwelling	0.88	1.02	0.73 – 1.41	0.96	1.15	0.94 – 1.41
**Toilet type**						
Flush type	1			1		
Pit/twin	1.12			1.33	1.63**	1.34 – 1.99
Open defecation	0.84			1.24	1.79**	1.33 – 2.40
**Cooking fuel**						
Clean	1			1		
Unclean	0.80			0.83*		
**Damp wall/ceiling**						
No	1			1	1	
Yes	1.17			0.66**	0.63**	0.51– 0.78

UOR: Unadjusted Odds Ratio; AOR: Adjusted Odds Ratio, * p value< 0.05** p value: < 0.01

Residence in an urban area, increasing income quintile, SC, OBC, and forward castes, households using unimproved toilet facilities have higher odds of CHIKV. Senior citizens (70 years and above), educated, kutcha houses, households with water-source in their own yard/plot and households with a damp wall/ceiling have lower odds of CHIKV. Urban residents have 1.8 times (AOR: 1.84; 95% CI: 1.36-2.49) higher likelihood of CHIKV than rural residents. The odds of CHIKV are highest in the richest (AOR: 1.92; 95%CI: 1.44-2.56) compared to the poorest. Respondents with primary school education (AOR: 0.80; 95% CI: 0.64-0.99), high school (6-12 grade) (AOR: 0.70; 95% CI: 0.57-0.86) and college education (AOR: 0.56; 95% CI: 0.38-0.85) have lower odds of CHIKV compared to those with no school education. All the other caste groups (SC, OBC and forward castes) have at least 1.5 times higher odds for CHIKV compared to the ST category. Households with improper toilet facilities like pit latrine/twin pit/composting toilet (AOR: 1.63; 95% CI: 1.34-1.99) and open defecation (AOR: 1.79; 95% CI: 1.33-2.40) have a higher likelihood of CHIKV. People residing in kutcha houses have lower odds of CHIKV (AOR: 0.50; 95% CI: 0.37-0.67) than those residing in pucca/semi pucca houses. Households with water-source in their yard/plot have lower odds (AOR: 0.57; 95% CI: 0.45-0.72) for CHIKV than households with water-source in their dwelling. Households with a damp wall/ceiling have lower odds (AOR: 0.63; 95% CI: 0.51-0.78) for CHIKV compared to those without a damp wall/ceiling.

## Discussion

Dengue and CHIKV infections are often mild and may be undiagnosed or misdiagnosed. Hence, we have only considered those who self-reported that they were treated for dengue or CHIKV. Dengue is the dominant vector-borne viral disease in India; population level serosurvey carried out in 2017-2018 (5-45 years) showed 48.7% seropositivity for dengue [7] vs. 18.1% for CHIKV [12]. Dengue is endemic in most states of India [5], and a population level serosurvey carried out in 2017-2018 in the age group of 5-45 years has reported seropositivity of 60.3%, 5%, 18.3%, 62.3% and 76.9% in the northern, north-eastern (NE), eastern, western and southern regions respectively [7]. The low self-reported prevalence could be due to the high seropositivity across India, except for the NE and eastern regions. The North Indian states of Delhi, Uttar Pradesh, Punjab and Haryana are the only ones to report ≥2% prevalence. Delhi is highly endemic for dengue, and multiple serotypes co-circulate [6]. Secondary infections resulting in severe dengue illness are known to occur due to the circulation of numerous serotypes [25] and may explain the highest self-reported prevalence (5.6%) in Delhi.

The high prevalence of CHIKV could be explained by the study period of the LASI survey. Even though the LASI survey was carried out in 2017-18, the respondents were asked to self-report if they had the disease in the preceding two years. In 2016, there was a massive outbreak of CHIKV in North India [26, 27]. The highest prevalence (>4%) of the self-reported CHIKV cases were in the northern states of Delhi, Uttar Pradesh, Haryana and Rajasthan. Even though the population level serosurvey shows South India to have the highest seropositivity (43.1%) [12], the self-reported cases in the LASI survey are lower. The southern states were the most affected in the CHIKV outbreak of 2005-06 [28–30]. A multicentric hospital-based study carried out in 2008-2009 to detect CHIKV cases by RT-PCR and/or IgM-ELISA reported the highest positive cases in South India (49.36%), followed by West (16.28%), and the lowest was in North (0.56%) [31]. Prior exposure to CHIKV could explain the low self-reported prevalence rates in the South when compared to North India. The eastern states of Odisha and West Bengal and the adjacent states of Bihar, Jharkhand and Chhattisgarh have <1% prevalence, and this overlaps well with the 4.4% seropositivity in the East [12]. Similarly, the prevalence was 0% in the North-East and is in line with the 0.3% seropositivity in this region [12]. In line with the population level serosurvey data [12], the LASI survey indicate eastern and the NE region of India to have low prevalence of CHIKV, and thus are susceptible to future outbreaks.

Analysis of LASI data indicates urban residence, wealth, low education, adults less than 55 years and location of water-source (water source in yard/plot has lower risk than water in the dwelling) to be the common risk factors for dengue and CHIKV in India. In addition, for CHIKV, caste (SC, OBC and forward), pucca/semi-pucca house type, unimproved toilet facilities are additional risk factors. Among the various factors of dengue transmission, urbanization, globalization and lack of effective vector control are considered to be the three major drivers [32]. *Ae. aegypti*, the primary driver of dengue and CHIKV is predominantly found in urban and peri-urban human habitation. In urban tropics, large swathes of human and *Ae. aegypti* population live in intimate association, and provide the perfect setting for the maintenance and generation of epidemic strains of vector-borne viruses [32, 33]. In this analysis, urban residence has higher odds for both dengue and CHIKV.

Positive association has been reported with dengue and CHIKV prevalence, and population density [34–38]. Even though dengue is present both in rural and urban India, incidence in urban areas is much higher; a nationwide dengue serosurvey has recorded 70.9% (64·3-76·6) seropositivity in urban compared to 42.3% (36·0-48·9) in rural districts [7]. The urban incidence of CHIKV is even higher; 40·2% (31·7-49·3) in urban *vs.* 11·5% (8·8-15·0) in rural [12]. *Ae. aegypti*’s breeding preferences coupled with population density makes urban areas a significant risk factor for vector-borne viral diseases in India. Among all the states and UT of India, the National Capital Territory of Delhi and the UT of Chandigarh are most urbanized with 97.5% and 97.25% urban population, respectively, followed by Daman and Diu at 75.2% [39]. Delhi shares borders with Haryana and Uttar Pradesh, and the urban expansion has accelerated in the border regions of these states [40]. Thus, this region has emerged as a hotspot of dengue and CHIKV in India. Even though Himachal Pradesh is bordering this hotspot region, the level of urbanization in Himachal Pradesh is least (10%) in the country, and this could explain the low period prevalence of dengue and CHIKV. Overall, urbanization appears to be a major driver of dengue and CHIKV.

Population based national serosurveys dengue and CHIKV incidence to increase with age; compared to 5-8 (Dengue: 28.3%; CHIKV: 9.2%) and 9-17 (Dengue: 41.0%; CHIKV: 14%), seropositivity is high in the 18-45 age group (Dengue: 56.2%; CHIKV: 21.6%) [7, 12]. The age group more susceptible to clinical dengue infection varies among different geographical regions, and is influenced by host immunity and the circulating viral genotypes. Epidemiology of the 2017 dengue outbreak in Sri Lanka show adults ≥ 50 years are least affected [41]. In Taiwan, dengue prevalence from 2010-2015 show significantly higher prevalence rates in adults ≥ 60 years [42]. Cyclical pattern of dengue epidemics driven by DENV-1 and DENV-2 serotypes have been observed in Singapore from 2004-2016; in DENV-2 predominant years (2007-12 and 2016), the incidence rate of dengue in 55+ age group is almost equal to the 15-24 years age group, while in DENV-1 predominant years (2004-2006 and 2013-2015), the incidence rate in 55+ years is about half [43]. In the 2007 epidemic in Brazil, there was a shift in the age pattern, with dengue hemorrhagic fever affecting predominantly children <15 (>53%), compared to 22.6% in 2001 [44]. For pan-India, reliable estimates of age-stratified dengue caseloads are not available in the public domain. A nine-year (2007-2015) dengue trend in Mumbai, western India, shows dengue morbidity to be highest in young adults aged 21-40 years [45]. Analysis of the LASI data among the three age groups (45-54, 55-69 and ≥70) shows adults in the 45-54 years age group to have higher odds for dengue. One possible reason for the higher likelihood in this group could be their active lifestyle related to employment, which would also make them travel frequently. A case-control study in Odisha, India, shows the odds of dengue are three times higher in individuals whose work requires long travel [17].

Location of water source outside the house was found to have slightly higher odds of both dengue and CHIKV though they were not significant. An individual-level cohort study carried out in Vietnam shows households that do not have access to tap water close to their dwelling have an increased risk of dengue fever [46]. Lack of access to piped water supply will lead to households resorting to using containers for water storage; these storage containers will provide the ideal breeding sites for mosquitoes resulting in increased dengue risk for the household [46]. A retrospective study carried out in Delhi has identified lack of access to tap water to be a key factor in dengue IgG seropositivity [15]. Lack of proper toilet facility in the household also increases the likelihood of CHIKV. *Ae. aegypti*’s peak biting periods are early in the morning, and in the period before dusk [47]; the need to use outside toilet facilities increases the likelihood of mosquito bites and vector-borne diseases.

Individuals with less than six years of schooling have higher odds of dengue and CHIKV. Several studies have shown the association between low education levels and dengue [48, 49]. Education helps in understanding the etiology of the disease, mode of transmission, symptoms, treatment, prevention and control measures [23]. Wealthy households have higher odds of dengue and CHIKV. Also, residents in pucca houses are more likely to get infected with CHIKV. Possible reasons include: 1) wealth is likely to be positively associated with urban residence; both dengue and CHIKV have a higher prevalence in densely populated urban settings in India, and 2) health-seeking behaviour may be better in wealthy households. In Delhi, the dengue burden was higher in wealthier districts despite a lower mosquito load [15]. In contrast, low SES is shown to be a key risk factor of dengue in Brazil [21, 22, 48, 50] and Cuba [51]. Unlike dengue hemorrhagic fever and dengue shock syndrome, dengue fever is self-limiting characterized by fever, myalgia, headache and constitutional systems [52]. The well-educated individuals from wealthy urban background are more likely to get diagnosed promptly compared to the lower socioeconomic class, and this may have increased the odds of dengue and CHIKV in the former. Future studies in different SES settings of India should be carried out to better understand the association between SES and dengue/CHIKV incidence.

Among the different social groups, ST have lower odds of CHIKV. The forest dominated Northeast (except Assam) and Central India states (Chhattisgarh, Jharkhand, Odisha, and Madhya Pradesh) have a high percentage (>20%) of ST [53], and malaria [54–57]. Except Madhya Pradesh (2.9%), the CHIKV prevalence is very low in all the other ST dominated states. Furthermore, the share of the ST population in urban areas is a meager (2.4%) and could be a key reason behind the lower odds in the ST [53].

The major limitation of the study is that the data analyzed to understand the socioeconomic and housing determinants are only from adults ≥ 45, therefore, it may not be appropriate to generalize these findings to all age groups. As the disease is self-reported, only respondents with symptomatic infection who got diagnosed may have reported, and this would affect the accuracy of the prevalence estimates. Furthermore, as LASI is a cross-sectional survey, the association of socioeconomic and household variables with dengue or CHIKV in this study does not imply causation.

## Conclusions

Dengue and chikungunya are two of the major vector-borne viral diseases that cause significant morbidity in India. For effective prevention and control strategies of dengue and chikungunya, it is important to understand the various social, economic and demographic risk factors that increase the odds of these infections in the population. Our analysis here shows in Indian adults aged 45 and above, both dengue and chikungunya are predominantly associated with urban settings. Among the factors that are associated with higher odds of dengue and CHIKV, improving education levels and better sanitation could be the focus of targeted interventions to reduce the prevalence.

## Supplementary Information

**Additional file 1.**

**Additional file 2.**

## Data Availability

The datasets can be obtained after submitting a data request form to IIPS, https://www.iipsindia.ac.in/content/lasi-publications.

## Abbreviations

CHIKV: Chikungunya
LASI: Longitudinal ageing study in India
IIPS: International Institute for Population Sciences
SES: Socio-economic status
SC: Scheduled castes
ST: Scheduled tribes
OBC: Other backward classes
SDH: Socio-economic determinants of health
