# Supplementary material for: Social and housing indicators of dengue and chikungunya in Indian adults aged 45 and above: Analysis of a nationally representative survey (2017-18)
Source: Arch Public Health. 2022 Apr 20;80:125. doi: 10.1186/s13690-022-00868-5 (PMC9022351; doi:10.1186/s13690-022-00868-5)
Supplement: Supplementary file 2 — Additional file 2. [file 13690_2022_868_MOESM2_ESM.pdf]

Point by point reply to reviewers' comments

On behalf of all the authors, I sincerely thank both the reviewers for their time and efforts spent on this manuscript. We have incorporated all the suggestions, and I have no doubt that the revised version is much better.

Our point-by-point comments to the reviewers' questions are addressed below

Reviewer#1: The reviewer has the following comments:
1. Please fully follow one of the EQUATOR Network Reporting Guidelines for the reporting of this study (e.g., the STROBE network). Please write in the manuscript that you have followed such statement.

Thank you for your suggestion. This study has followed the STROBE guidelines, and we have mentioned it in statistical analysis under the methods section

2. Please fully follow the guidelines for the submission of this manuscript to the journal as some sections are missing or should not be reported.

Thank you for the suggestion.

Accordingly, we have made the following edits.

- Author summary has been removed
- Limitation has been now moved to the discussion section (last paragraph) from the conclusion section
- Pages are numbered
- Figures-caption and legend have been included
- Tables-size have been adjusted to fit the journal requirement

Reviewer #2: This study used data from a nationally representative survey to examine the association between socioeconomic factors and self-reported dengue and chikungunya prevalence. The authors found that urban residency, education level, wealth status, toilet or house type are significant factors for both diseases. Overall, the manuscript is well written, methods are described in adequate detail, statistical methods are sound, and the discussion has covered most aspect of the results in the context of the literature and dengue/chikungunya situation in other countries. Nevertheless, the manuscript can be further improved with the following points:

Due to the self-report nature of the study, only considering individuals treated by health professionals may bias the results to show higher odds for those who are wealthy and highly educated. This is discussed briefly in the discussion. However, I recommend a sensitivity analysis on those who self-reported having dengue or chikungunya in the past two years. The sensitivity analysis will verify whether the positive associations for the wealthiest/highly educated/forward castes still hold.

Thank you for your suggestion**.** Dengue and CHIKV infections are often mild and mostly go undiagnosed or misdiagnosed. Hence, we have only considered those who had self-reported dengue/CHIKV infection and were also treated for these two diseases. The self-reported dengue cases were 674; of those, 607 (91%) got treated by a health professional, CHIKV cases were 1427, and 1358 (95%) were treated by a health professional. Most (>90%) of the self-reported cases got treated, which implies that they are indeed cases of dengue/CHIKV. A separate analysis for the actual self-reported cases with predictors and what the authors have considered (who had self-reported dengue/CHIKV infection and also got treated for these two diseases) and the predictors were performed. There was hardly any difference in the analyses except for slight variations in values. The positive associations for the wealthiest/highly educated/forward castes did not change after the sensitivity analysis.

Throughout the manuscript, avoid stating that certain factors "increase" the odds of dengue/chikungunya since the analysis are only meant for identifying "association" between factors and outcome, but not identifying causality relationship

Thank you for this important suggestion. We have modified accordingly. Furthermore, we have replaced determinants in the title with indicators.

"Social and housing ~~determinants~~ indicators of dengue and chikungunya in Indian adults aged 45 and above: Analysis of a nationally representative survey (2017-18)"

Why not mentioning urban residency and age as important factors in the abstract?

We have added a statement of these two variables in the abstract**.**

Line 38, [estimated] instead of calculated

Done

Line 102, should mention the species are mosquito species

Done

Line 104, epidemiological spread [of dengue]

Done

Line 107, dengue risk is [reported] in 129 countries

Done

Methods should briefly explain the sampling design. Did they sample within state/urban-rural setting? Is it nationally representative?

Again, we appreciate this important suggestion and the paragraph below is included in the methods section.

"The LASI wave 1 is a nationally representative study of all states and Union Territories (UT)except Sikkim. The survey gathered vital information on health, infectious diseases, socioeconomic determinants, and consequences of population ageing from 72,252 individuals. A multistage clustering sampling design was adopted to obtain the data from the non-institutional residents aged ≥45 years and their spouses (regardless of age). In the first stage of the sampling process, primary sampling units, i.e., sub-districts (Tehsils/Talukas), were selected in each state/UT. During the second stage, villages (rural areas) and wards (urban areas) were selected from all the primary sampling units and in the third stage, households and individuals were selected. The sampling procedure was extended by one more step in the urban areas where a census enumeration block was chosen before selecting households"

Line 170, why did you exclude the untreated respondents from the analysis, since you can consider them as not having the two diseases but still kept them in the model?

The sample size earlier was 70,865, and now it is 70,932 after including the untreated respondents. The analysis was repeated again with 70,932 respondents.

Line 177 should consider separating toilet-type into three groups: flushed types, pit/latrine/twin pit/composting toilet, and open defecation.

As suggested, the toilet-type was coded into three types: flushed types, pit/latrine/twin pit/composting toilet, and open defecation, and we have re-run the analysis. Now, we find pit latrine/twin pit/composting toilet and open defecation to be associated with CHIKV.

Line 207, accurate [statistical] estimates

Done

Line 207-209 should be introduced when you described the data and variables before statistical methods.

Thank you. The lines have been moved to the heading "data and participants"

Line 210, any patterns for those with missing values?

We have checked and have not observed any patterns for the missing value. The missing values are less than 2%.

Line 210, reference for Stata is required

Done

Line 215, which version of ArcGIS is used? Reference is required.

We have used ArcGIS 10.4, and have added the reference link

Line 216, why using average? You can obtain the national prevalence as the mid-point instead

We have considered mid-points and natural breaks in the datasets while determining class intervals for spatial representation. We have changed the statement to remove ambiguity

Line 233, are the prevalence for dengue or chikungunya?

We have edited the statement

Conclusion should provide policy implications of the results

Briefly, we have written the policy implications in the conclusions.

Please check wordings: Line 244, [non-]working individuals; Line 245, rural [residents]; Line 248, [lower] odds; Line 252, [higher] odds; and elsewhere

Done
